# Supplementary material for: Systematic Review of Prehospital Prediction Models for Identifying Intracerebral Haemorrhage in Suspected Stroke Patients
Source: Healthcare (Basel). 2025 Apr 11;13(8):876. doi: 10.3390/healthcare13080876 (PMC12026686; doi:10.3390/healthcare13080876)
Supplement: Supplementary file 1 [file healthcare-13-00876-s001.zip › Supplementary File S2.pdf]

Supplementary File S2. Search strategies for the systematic review.

Database searched: MEDLINE (Ovid)

#	Query
1	exp Brain Ischemia/ or exp Stroke/ or exp Hemorrhagic Stroke/ or exp Cerebral Hemorrhage/ or exp Intracranial Hemorrhages/
2	Intracerebral hemorrhage.mp.
3	1 or 2
4	exp cerebrovascular accident/di
5	exp stroke/di
6	4 or 5
7	(Predict$ or Model$ or Decision$ or Identif$ or Prognos$).mp.
8	3 and 7
9	6 or 8
10	exp Emergency Medical Services/ or Paramedic*.mp. or ambulance.mp. or triage.mp. or prehospital.mp. or pre hospital.mp. or EMS.mp. or emergency medical service*.mp.
11	Air Ambulances/
12	Emergency Service, Hospital/
13	Emergency Medicine/
14	10 or 11 or 12 or 13
15	9 and 14
16	limit 15 to english language

Database searched: EMBASE (Ovid)

#	Query
1	exp Brain Ischemia/ or exp Stroke/ or exp Hemorrhagic Stroke/ or exp Cerebral Hemorrhage/ or exp Intracranial Hemorrhages/
2	Intracerebral hemorrhage.mp.
3	1 or 2
4	exp cerebrovascular accident/di [Diagnosis]
5	exp stroke/di
6	4 or 5
7	(recogni$ or identi$ or strati$ or distinguish$ or probabilit$).mp.
8	3 and 7
9	6 or 8
10	exp Emergency Medical Services/ or Paramedic*.mp. or ambulance.mp. or triage.mp. or prehospital.mp. or pre hospital.mp. or EMS.mp. or emergency medical service*.mp. or out-of-hospital.mp.
11	Air Ambulances/
12	Emergency Service, Hospital/
13	10 or 11 or 12
14	9 and 13
15	limit 14 to english language

Database searched: CENTRAL (Ovid)

#	Query
1	exp Brain Ischemia/ or exp Stroke/ or exp Hemorrhagic Stroke/ or exp Cerebral Hemorrhage/ or exp Intracranial Hemorrhages/
2	Intracerebral hemorrhage.mp.
3	1 or 2
4	exp cerebrovascular accident/di
5	exp stroke/di
6	4 or 5
7	(Predict$ or Model$ or Decision$ or Identif$ or Prognos$).mp.
8	3 and 7
9	6 or 8
10	exp Emergency Medical Services/ or Paramedic*.mp. or ambulance.mp. or triage.mp. or prehospital.mp. or pre hospital.mp. or EMS.mp. or emergency medical service*.mp.
11	Air Ambulances/
12	Emergency Service, Hospital/
13	Emergency Medicine/
14	10 or 11 or 12 or 13
15	9 and 14
16	limit 15 to english language
