# Supplementary material for: Systematic Review of Prehospital Prediction Models for Identifying Intracerebral Haemorrhage in Suspected Stroke Patients
Source: Healthcare (Basel). 2025 Apr 11;13(8):876. doi: 10.3390/healthcare13080876 (PMC12026686; doi:10.3390/healthcare13080876)
Supplement: Supplementary file 1 [file healthcare-13-00876-s001.zip › Supplementary File S3.pdf]

Table S1. Predictors considered in each of the nine prediction modelling studies.

**Predictors**	**Woisetschläger 2000**	**Yamashita 2011**	**Jin 2016**	**Uchida 2018**	**Chiquete 2021**	**Uchida 2021**	**Geisler 2021**	**Hayashi 2021**	**Uchida 2022**
**Patient demographics**									
Age	Δ	Δ	✓	X	X	X	X	✓	✓
Sex	Δ	Δ	X	X	X	X	X	✓	✓
**Medical history**									
DM	✓	X	✓	-	X	-	-	✓	-
HTN	✓	X	✓	-	✓	-	X	✓	-
Hyperlipidaemia	Δ	Δ	-	-	-	-	-	-	-
Current smoking	Δ	X	-	Δ	-	Δ	-	-	X
Prior MI or coronary artery disease	Δ	Δ	-	-	-	-	-	-	-
AF	Δ	✓	✓	-	X	-	X	✓	-
Prior use of anticoagulation or antiplatelet agents	Δ	Δ	-	Δ	-	X	-	✓	-
ICH	-	Δ	-	Δ	X	X	-	✓	-
IS or TIA	-	X	-	✓	X	Δ	-	✓	X
SAH	-	-	-	X	X	X	-	-	-
**Signs and symptoms**									
Headache	✓	Δ	-	✓	✓	✓	-	✓	✓
Dizziness	-	-	-	Δ	-	Δ	-	✓	✓
Speech or language deficit	✓	Δ	-	✓	✓	✓	Δ	✓	✓
Nausea or vomiting	✓	Δ	✓	X	✓	X	-	✓	✓
Seizure or convulsion	✓	Δ	-	X	-	X	X	✓	✓
Impaired LOC	✓	✓	X	✓	✓	✓	✓	✓	✓
Focal or unilateral weakness	✓	Δ	-	-	✓	-	✓	✓	-
Numbness or sensory loss	✓	Δ	-	X	-	X	✓	✓	-
Gaze deviation	-	Δ	-	✓	-	✓	Δ	✓	✓
Visual field defect	-	X	-	-	-	-	✓	✓	-
Anisocoria	-	-	-	Δ	-	X	-	-	-
Unilateral spatial neglect	-	X	-	Δ	-	Δ	Δ	✓	X
Facial palsy	-	X	-	Δ	-	Δ	Δ	✓	✓
Weakness or paralysis of limbs	-	Δ	-	✓	-	✓	✓	✓	✓
Sudden onset	-	-	-	Δ	-	Δ	-	-	✓
Symptoms improved after onset	-	-	-	✓	-	Δ	-	-	✓
Symptoms progressed after onset	-	-	-	✓	-	Δ	-	-	✓
Items of the NIHSS	-	✓	-	-	-	-	✓	-	-
**Vital signs**									
SBP	Δ	Δ	✓	✓	-	✓	✓	✓	✓
DBP	Δ	✓	Δ	✓	-	Δ	X	✓	✓
HR	-	-	Δ	-	-	-	-	✓	-
Arrhythmia	-	-	Δ	✓	-	✓	-	✓	✓

(✓): included in the final model.

(Δ): excluded during modelling.

(X): excluded before modelling.

(-): not measured/collected.

Abbreviations: AF, atrial fibrillation; DBP, diastolic blood pressure; DM, diabetes mellitus; HR, heart rate; HTN, hypertension; ICH, intracerebral haemorrhage; IS, ischaemic stroke; LOC, level of consciousness; MI, myocardial infarction; NIHSS, National Institutes of Health Stroke Scale; SAH, subarachnoid haemorrhage; SBP, systolic blood pressure; TIA, transient ischaemic attack.

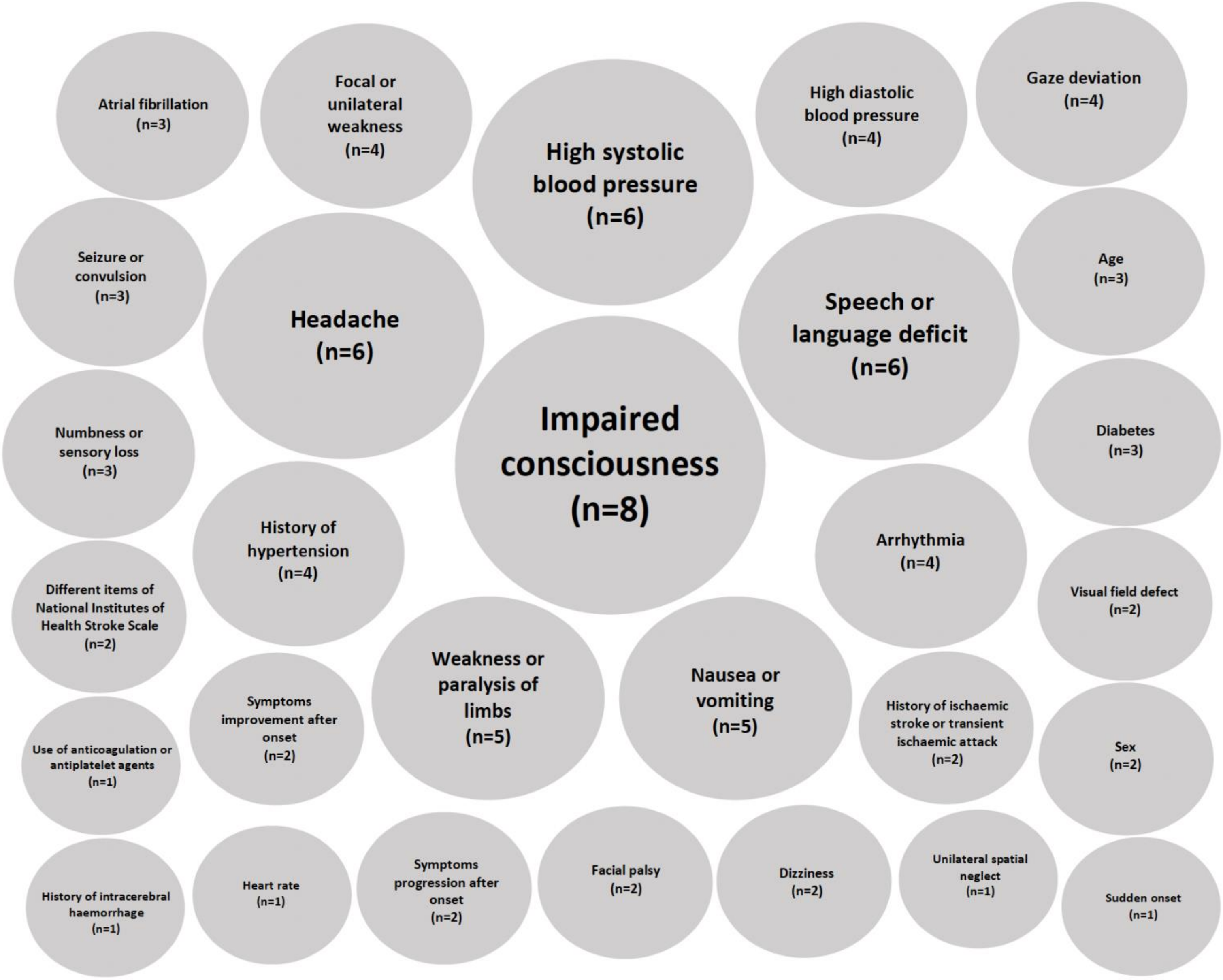

Figure S1. Frequency of identified predictors in the final prediction models.
